# H_2_O_2_ as a Feedback Signal on Dual-Located WHIRLY1 Associates with Leaf Senescence in *Arabidopsis*

**DOI:** 10.3390/cells8121585

**Published:** 2019-12-06

**Authors:** Wenfang Lin, Dongmei Huang, Ximiao Shi, Ban Deng, Yujun Ren, Wenxiong Lin, Ying Miao

**Affiliations:** Fujian Provincial Key Laboratory of Plant Functional Biology, College of Life Sciences, Fujian Agriculture and Forestry University, Fuzhou 350002, China; linwf@fafu.edu.cn (W.L.); 2130516002@fafu.edu.cn (D.H.); 1160539005@fafu.edu.cn (X.S.); 1140539002@fafu.edu.cn (B.D.); ryj@fafu.edu.cn (Y.R.); wenxiong181@163.com (W.L.)

**Keywords:** WHIRLY1 (WHY1), H_2_O_2_, histone lysine modification, dual-location, plant senescence, *Arabidopsis thaliana*

## Abstract

Leaf senescence, either as a natural stage of development or as an induced process under stress conditions, incorporates multiple intricate signaling pathways. At the cellular level, retrograde signals have been considered as important players during the initiation and progression of senescence in both animals and plants. The plant-specific single-strand DNA-binding protein WHIRLY1 (WHY1), a repressor of leaf natural senescence, is dually located in both nucleus and plastids. Despite many years of studies, the myth about its dual location and the underlying functional implications remain elusive. Here, we provide further evidence in *Arabidopsis* showing that alteration in WHY1 allocation between the nucleus and chloroplast causes perturbation in H_2_O_2_ homeostasis, resulting in adverse plant senescence phenotypes. The knockout of WHY1 increased H_2_O_2_ content at 37 days post-germination, coincident with an early leaf senescence phenotype, which can be rescued by ectopic expression of the nuclear isoform (nWHY1), but not by the plastid isoform (pWHY1). Instead, accumulated pWHY1 greatly provoked H_2_O_2_ in cells. On the other hand, exogenous H_2_O_2_ treatment induced a substantial plastid accumulation of WHY1 proteins and at the same time reduced the nuclear isoforms. This H_2_O_2_-induced loss of nucleus WHY1 isoform was accompanied by enhanced enrichments of histone H3 lysine 9 acetylation (H3K9ac) and recruitment of RNA polymerase II (RNAP II) globally, and specifically at the promoter of the senescence-related transcription factor *WRKY53*, which in turn activated *WRKY53* transcription and led to a senescence phenotype. Thus, the distribution of WHY1 organelle isoforms and the feedback of H_2_O_2_ intervene in a circularly integrated regulatory network during plant senescence in *Arabidopsis*.

## 1. Introduction

Organelles, especially plastids, mitochondria, and peroxisomes, are considered as the sensors for cellular stress signal perception, and the generated signals are usually transduced to the nucleus leading to the occurrence of nuclear events, a process referred to as retrograde signaling [1,2,3]. For example, several retrograde signaling molecules, such as O_2_ and H_2_O_2_ from plastids, evoke regulatory information to the cytosol and nucleus via downstream messengers and/or a complex signaling network [4,5]. Other plastid signal molecules, including 3′-phosphoadenosine 5Ȳ-phosphate (PAP, a phosphonucleotide) [6], methylerythritol cyclodiphosphate (MEcPP, an isoprenoid intermediate) [7], heme [8,9], and malate [10], are also reported. In other cases, a signal transduction may be trigged via the movement of proteins from organelles to the nucleus, especially those of the membrane-bound proteins or dual-targeting transcription factors. Among them, the transmembrane domain-containing PHD type transcription factor (PTM), the plastid envelope DNA-binding protein (PEND), and the dual-located single-stranded DNA-binding protein WHIRLY1 are known examples [4,5]. The PTM is released from the chloroplast envelope through proteolytic cleavage and transmits multiple retrograde plastid signals to the nucleus by activating the ABSCISIC ACID INSENSITIVE 4 (ABI4) transcription factor [11]. Moreover, transcription factors from the APETALA2/ethylene-responsive element binding protein family (AP2/EREBP) are involved in retrograde signaling by integrating metabolic, hormonal, and environmental signals [12], and the GOLDEN2-LIKE transcription factors (GKLs) participate in activating retrograde immunity signals in response to phytochrome B (PhyB) [13,14,15,16].

The dually located WHIRLY proteins have three members (WHY1, WHY2, and WHY3) in most dicotyledons, and WHY3 is not found in monocotyledons. All the three proteins were found to be located in nucleus, as well as in plastids (WHY1 and the somehow redundant WHY3) or mitochondria (WHY2). They were shown to perform several cellular functions at both locations [17,18,19,20]. In the nucleus, WHIRLY proteins were found to regulate the expression of genes related to defense and senescence by binding at their promoters [21,22]. The WHY1 protein bound to the promoter of *WRKY53* in a development-dependent manner during early senescence in *Arabidopsis* [23], while in barley the ortholog could activate the *HvS40* gene during natural and stress-related senescence [24]. In tomato, ortholog WHY1 regulated the *SlPsbA* gene in response to chilling treatment [25]. The involvement of WHY1 protein in modulating telomere length by binding to the AT-rich region of telomeres has also been suggested [26]. We recently found that Arabidopsis WHY1 accumulation in the nucleus altered the enrichment of di/trimethylation of histone H3 at lysine 4 (H3K4me2/3) and H3K9ac and the recruitment of RNA polymerase II (RNAP II) at the promoter’s AT-rich region of *WRKY53*, repressing *WRKY53* transcription [27]. 

In plastids, the WHY1 protein is located at the boundary between thylakoids and nucleoids. This plastid isoform could be detected in nucleoids either as part of the so-called transcript active chromosome (TAC) components [28,29] or as an RNA-binding protein [30], with the latter suggested to function in organelle genome stability via assisting accurate DNA repair [31,32,33]. In addition, WHY1 association with intron-containing RNA was also observed, hinting at a role in intron splicing in the chloroplasts [29,30]. Under stress conditions, WHY1 might also be involved in chloroplast photosynthetic redox sensing by producing retrograde signals to the nucleus [19,34,35]. The knockdown of *WHY1* in barley led to reprogramming of genes encoding chloroplast proteins and a decline in photosynthetic sensitivity to low-nitrogen conditions, an outcome which might be attributed to the disruption of communication between the plastids and the nucleus [36]. A simultaneous loss of *WHY1*, *WHY3*, and the chloroplast DNA polymerase 1B (*Pol1B*) produced an acute yellow-variegated phenotype, correlating with significant expression changes in numerous oxidation-related nuclear genes [33]. Moreover, the *why1why3polIb-1* mutant line showed decreased photosynthetic electron transport (PET) efficiency and enhanced accumulation of reactive oxygen species (ROS) compared to wild-type plants [33]. It seemed that both isoforms and locations of WHY1 were critical for its roles in senescence repression or might be relevant to retrograde signaling; however, it was unclear how the dual localization was regulated. 

We have previously shown that the nuclear isoform WHY1 is required for delayed leaf senescence via its repression of senescence-related genes, such as *WRKY53*, *Senescence Associated Gene 12* (*SAG12)*, and *NADH dehydrogenase F* (*NDHF)*; and that the accumulation of this nuclear isoform depended on phosphorylation of the proteins by Calcineurin B-Like-Interacting Protein Kinase14 (CIPK14), a SNF1-related protein kinase [20,23]. Furthermore, the plastid isoform of WHY1 interacted with the light-harvesting protein complex I member (LHCA1), and the loss-of-function mutant *why1* was compromised in both gene expression and functionality for photosystem I (PSI) and light-harvesting complexes (LHCI) [37]. The ectopic overexpression of the plastid isoform of WHY1 did not truly reverse the loss-of-function mutation, indicating a tight control of proper allocation under normal physiological conditions, possibly by a global signaling pathway linking the chloroplast’s function and nuclear regulation [37]. Here, we found that the plastid isoform of WHY1 had a negative effect on H_2_O_2_ homeostasis, while the nuclear isoform of WHY1 was required for balancing the homeostasis of H_2_O_2_ in the cells. H_2_O_2_ treatment reduced the distribution of WHY1 proteins in the nucleus, but increased the plastid isoforms. Our results demonstrated the involvement of H_2_O_2_ in regulating the allocation of WHY1 between the nucleus and chloroplast, with respective to leaf senescence in *Arabidopsis*.

## 2. Materials and Methods

### 2.1. Plant Materials and Culture Conditions

Wild-type plants of *Arabidopsis thaliana* (L.) Heynold ecotype Columbia (WT) were used. The T-DNA insertion lines *why1* (Salk_023713) [23], *peroxidase 39* (*prx39)* (SAIL_757_G03), and *peroxidase 33* (*prx33)* (SALK_062314) were obtained from the European Arabidopsis Stock Centre, and the homozygous plants were selected and confirmed by PCR and RT-PCR using genomic DNA (gDNA) and mRNA as templates, respectively, with primers listed in Supplementary Table S1. The antisense WHY1 (*awhy1*) line (*35S:WHY1RNAi*) has been reported previously [23]. 

To generate transgenic plants overexpressing WHY1 organelle isoforms differently localized in the nucleus or dually located in plastids and the nucleus, the plasmids *nWHY1-HA*, *pnWHY1-HA,* and WHY1 own promoter driving WHY1 coding sequence plus HA tag *Pwhy1:pnWHY1-HA* (*PWHY1-HA)* were used, as described in a previous study [23]. The *pWHY1-HA* plasmid, kindly provided by Krupinska’s lab, harbored the construct of the full-length WHY1 plus the nuclear export peptide sequence fused to an hemagglutinin (HA) tag for producing WHY1 only in plastids [19]. All cassettes were sub-cloned into binary vectors driven by the 35S promoter [20,23]. All these overexpression lines were constructed in the *why1* background and the transgenic plants were selected by spraying 0.1% (*w*/*v*) glufosinate-ammonium (Basta, Bayer Crop Science, Germany). Homozygous transgenic plants were obtained at the third generation (T3). The expression of *WHY1* in these mutants were monitored by both quantitative RT-PCR and immunoblot detection using an antibody against the HA tag (Supplementary Figure S1).

Seedlings germinated on wet filter paper were subjected to vernalization at 4 °C for 2 d, then transplanted to vermiculite and maintained in a climatic chamber (100 μE/h, 13 h of light at 22 °C/11 h of dark at 18 °C, under 60% relative humidity). The rosette leaves were labeled with colored threads after emergence, as described previously [38]. 

For H_2_O_2_ treatments, plants were kept without watering for two days before spraying with 0.3% hydrogen peroxide solution. Rosette leaves were collected at 1,4,6, and 8 h after sprays and stored in liquid nitrogen or at −80 °C for later use in RNA or protein isolations. Mock treatments used distilled water.

### 2.2. Chlorophyll Fluorescence and Concentration Measurement

For chlorophyll fluorescence and concentration measurement, the seventh leaf from individual plants at different stages of development was sampled. After dark incubation for 15 min, chlorophyll fluorescence was measured at three spots on each leaf taken from at least 12 individual plants using a Pocket PEA chlorophyll fluorimeter (Hansatech Instruments, Norfolk, UK). Chlorophyll concentration was determined with Dualex 4 (FORCE-A, Paris, France). Data were shown as mean ± SD.

### 2.3. Measurement of H_2_O_2_ Content in Rosette Leaves

Quantitative H_2_O_2_ measurements were made using the Amplex Red Hydrogen Peroxide/Peroxidase Assay Kit (Molecular Probes, Thermo Fisher China, Shanghai, China) following the manufacturer’s instructions. Briefly, leaves were ground into fine powders in liquid nitrogen and 30 mg of the powders was suspended in 200 μL of the extraction buffer (25 mM Tris-HCl buffer, pH 6.5). The supernatant was collected after centrifugation at 12,000 rpm for 15 min at 4 °C and used for the quantitative assay. The measurement of 560 nm absorbance was performed using Tecan Infinite F200/M200 (Tecan, Männedorf, Switzerland) equipped with a microplate reader (FlexStation3, Molecular Devices, San Jose, Unite states). H_2_O_2_ concentration was calculated based on the fresh weight of the leaves used. 

### 2.4. Staining of Hydrogen Peroxide

The visualization of H_2_O_2_ accumulation in leaves was performed using the 3′,3′-diaminobenzidine (DAB) staining method according to Yang et al. (2014) and Huang et al. (2019) [39,40]. Detached rosette leaves were vacuum filtered in 20 mL staining solution containing 1 mg/mL DAB in 50 mM Tris-HCl, pH 5.0 for 10 min, and incubated in darkness at room temperature for 12 h. The stained leaves were clarified by boiling in a mixture of ethanol, glycerol, and acetic acid (3:1:1, *v*/*v*/*v*) for 15 min [41] before imaging.

The detection of superoxide free radicals was performed by the nitro blue tetrazolium (NBT) staining method described by Lee et al. (2002) [42]. The whole rosette leaves of 5- to 6-week-old plants were harvested and immersed in 0.1 mg/mL of NBT solution (25 mM HEPES, pH 7.6). After vacuum infiltration, samples were incubated at 25 °C for 2 h in the darkness. Subsequently, stained samples were bleached in 70% ethanol and incubated further for 24 h at 25 °C to remove the chlorophyll.

Imaging was conducted using an Epson Perfection V600 Photo scanner (Epson China, Beijing, China).

### 2.5. Quantitative RT-PCR Analysis (qRT-PCR)

The qRT-PCR analysis was performed using the SYBR Green master mix (SABiosciences, Frederick, MD, USA) according to the manufacturer’s instructions. Complementary DNA synthesis was carried out using the Fermentas First Strand cDNA Synthesis Kit (Thermo Fisher Scientific, Waltham, MA, USA) on RNA from 28- to 55-day-old plants grown under normal light conditions. Complementary DNAs were diluted 20-fold prior to quantitative PCR experiments. The Touch 1000 platform (Bio-Rad Company, Beijing, China) was used for qRT-PCR experiments, and the data were analyzed using the Bio-Rad software version 1.5. We used *glyceraldehyde-3-phosphate dehydrogenase C2* (*GAPC2)* or *ACTIN2* (for monitoring gene expression in plants, including the *wrky53* plants) as the internal reference gene for calculation of relative expression. Primers are listed in Supplementary Table S1. All determinations were conducted in three biological replicates.

### 2.6. In Vitro DNA-Binding Assays

Recombinant WRKY53 and WRKY33 proteins were produced in *Escherichia coli* as described by Miao et al. [23,43]. An electrophoretic mobility shift assay (EMSA) was performed following the protocol in the same reference. The DNA probes were amplified from *Arabidopsis* gDNA by using specific primer pairs listed in Supplementary Table S1. Labeling of the probes with ^32^P was achieved using the 5′-end labeling protocol with T4 polynucleotide kinase forward reaction.

### 2.7. Isolation and Detection of Plastid and Nuclear Proteins

Chloroplasts were prepared and purified on Percoll gradients as described in our previous paper [20]. A detailed protocol for nuclei isolation could be found in the same reference. Approximately 8 μg protein of each fraction was separated on 14% (*w*/*v*) acrylamide gels. After transferring to nitrocellulose membranes, immunodetection was performed using specific antibodies against the WHY1 C-terminal peptide, CASPNYGGDYEWNR (Faan, Hangzhou, China). To monitor the purity of the chloroplast and nuclear fractions, we used antibodies against cytochrome b559 apoprotein A [44] and histone H3 (Cell Signaling, Munich, Germany), respectively (Supplementary Figure S5). 

### 2.8. ChIP-qPCR Assay

Chromatin immunoprecipitation (ChIP) assays were performed using 1.5 g of leaf tissue from entire rosettes of 4-week-old plants at 4 h after H_2_O_2_ treatment, as described in a previous paper [23]. Antibodies against H3K4me2 (Cat. Nr. 07-030, Upstate Biotechnology Inc, Lake Placid, NY, USA), H3K4me3 (Cat. Nr. 07-473, Upstate Biotechnology), H3K9ac (Cat. Nr. 07-352, Upstate Biotechnology), histone H4 acetylation (H4ac) (Cat. Nr. 07-352, upstate Biotechnology), and RNAP II (Cat. Nr. ab817, Abcam, Cambridge, GB, USA) were used to immunoprecipitate genomic DNA. After purification, the precipitated DNAs were used as templates for qPCR to determine the enrichment of two fragments in the *WRKY53* promoter, one encompassing the GTNNNAAAT motif region (−416 to −266 upstream of transcription start site (TSS) and the other flanking the 5′-end untranslated region (UTR) and first exon region (−155 to +77). The primers can be found in Supplementary Table S1. Relative enrichment was calculated by the ChIP/input ratio and then normalized to H_2_O treatment to obtain fold change over the mock. The assays were conducted for three biological replicates.

For ChIP-qPCR determinations of WHY1-HA or WRKY53-HA occupancies on promoter regions in *PRX39* or *PRX33* gene, four-week-old rosettes of transgenic plants of the WHY1-HA-overexpressing line (*why1* background) or the WHRY53-HA-overexpressing line (WT background) were used in sample preparations, respectively. The cross-linked DNA fragments ranging from 200 to 1000 bp in length were immunoprecipitated by an antibody against the HA tag (Cell Signaling, Munich, Germany). The enrichment of the selected promoter regions of both genes was resolved by comparing the amounts in the precipitated and non-precipitated (input) DNA samples, which were quantified by quantitative PCR using designed region-specific primers (Supplementary Table S1). The same quantification in mutant line *why1* or WT served as a control for the respective overexpression lines, and was used for normalizations to give the fold enrichment factors over the mock. The experiments were performed in three biological replicates.

### 2.9. Statistical Analysis

Where appropriate, quantitative data were determined by at least three biological replicates and the statistical significance was analyzed either using two-way ANOVA or pair-wide multiple t-tests, with the GraphPad Prism software version 7 (GraphPad Software, San Diego, CA, USA).

## 3. Results

### 3.1. Ectopic Expression of a Plastid Isoform WHY1 Causes a Strong Leaf Senescence 

**Figure 1:**
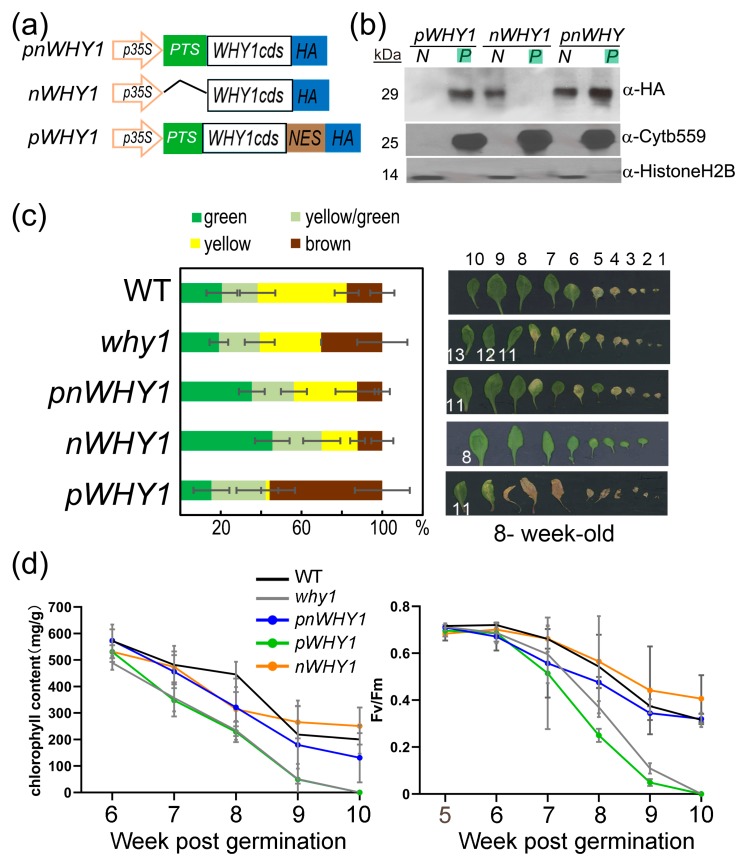
Ectopic overexpression of differently localized WHY1 isoforms in the *why1* background. (**a**) Schematic constructs for 35S promoter-driven expression of WHY1 isoforms: pnWHY1, the full-length WHY1 coding sequence (CDS) plus an HA tag; nWHY1, the WHY1 CDS minus the plastid transit signal (PTS) plus the HA tag; and pWHY1, the full-length WHY1 CDS plus a nuclear export signal (NES) and the HA tag; (**b**) Western blot images showing subcellular (N: nucleus, P: plastids) accumulation of WHY1 isoforms in leaves of respective transgenic plants, using antibodies against the HA tag, histone H_2_B, and cytochrome b559 (cytb559); (**c**) Left, statistic assessment of senescent leaves by color at the sixth week post-germination, error bars represent the SD for 30 plants each. Right, visualization of leaves of a typical plant at the same age, leaves were ordered by emergence; (**d**) Chlorophyll content and photosystem II fluorescence index (Fv/Fm) of the seventh rosette leaf measured after the fifth week post-germination. Error bars indicate the SD of five independent measurements.

To address the subcellular functions of WHY1, we generated transgenic lines overexpressing different organelle isoforms of WHY1 in the *why1* background [20,23,37]. These included the plastid isoform of WHY1 (pWHY1), which contained the full-length WHY1 coding sequence plus a nuclear export peptide; the nuclear isoform WHY1 (nWHY1), which contained the WHY1 coding sequence without its plastid transit peptide; and the full-length WHY1 (pnWHY1) (Figure 1a; Supplementary Figure S1). Homozygous transgenic lines obtained after screening for the third generation were used to compare subcellular localization of the expressed WHY1 proteins in leaf preparations by western blot (Figure 1b). As expected, expressed pWHY1 and nWHY1 were predominantly detected in plastids and nucleus, respectively, while pnWHY1 gave signals in both the nucleus and plastids (Figure 1b). The *why1* plants were early senescent when compared with WT, whereas overexpression of the full-length WHY1 (pnWHY1) or the nuclear isoform nWHY1 could rescue this phenotype or delay leaf senescence (Figure 1c,d). These phenotypes were consistent with previous observations [20,23,37]. The overexpression of the plastid isoform of WHY1 (pWHY1) resulted in an accelerated senescence phenotype—apparently pale yellow or cell death in rosette—even more pronounced than *why1*, as justified by parameters including the total ratio index of leaf coloring, chlorophyll content, and photosystem II fluorescence index (Fv/Fm, ratio of variable fluorescence to maximum fluorescence), of the seventh leaf (Figure 1c,d). Typically, differential senescence phenotypes began during the sixth week (approximately at 35 to 42 days post-germination, Figure 1c,d). In this period, WT exhibited peak expression levels of *WRKY53* and an activated transcription of *SENESCENCE-ASSOCIATED GENE 12* (*SAG12*) (Supplementary Figure S2a).

**Figure 2:**
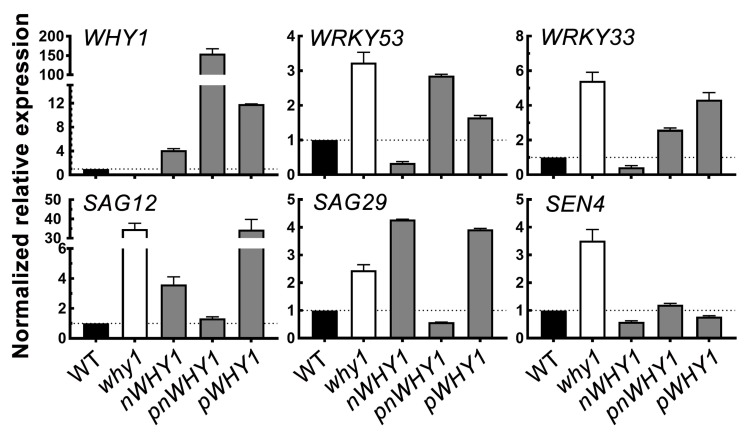
Relative mRNA levels of senescence-related genes in leaves of six-week-old wildtype and *why1* transgenic lines. Reverse transcription quantitative PCR using *GAPC* as the reference gene was conducted and data were normalized to that of WT. The error bars represent SD from three biological replicates.

At this developmental stage, the steady-state gene expression was monitored and compared in these overexpression lines. The selected genes included several senescence-related genes, including *WRKY53*, *WRKY33*, *SAG12*, *SAG29 (SENESCENCE-ASSOCIATED GENE 29)* and *SEN4 (SENESCENCE 4*). In consistence with the phenotypes, the five genes were upregulated in the loss-of-function *why1* mutant. Among the three transgenic plant lines overexpressing different *WHY1* isoforms, however, abnormal *WHY1* transcript accumulation and discrepancy between gene expression and phenotypes were observed (Figure 2). The *nWHY1* plant, which accumulated approximately 4-fold higher *WHY1* transcripts over WT plants, had apparently lower expression levels for its repressed targets *WRKY53* and *WRKY33*, as well as *SEN4*. But on the other hand, this line expressed significantly higher *SAG12* and *SAG29* (Figure 2), which encoded, respectively, a cysteine protease and a sucrose transporter and functioned in remobilization of nitrogen and carbon in senescent organs as well as in normal organs under stress conditions [45,46,47]. This line showed more or less delayed leaf senescence as compared to the *why1* mutant or WT (Figure 1). Furthermore, in the *pWHY1* transgenic line that accumulated approximately 11.9-fold more *WHY1* transcripts than the WT, only the expression of *SEN4* and to some extent, the expression of *WRKY53* were restored to the WT level, while transcripts of *WRKY33*, *SAG12*, and *SAG29* were higher than in WT by a factor of about 4, 34, and 4, respectively (Figure 2). The *pnWHY1* transgenic line displayed the highest level of *WHY1* transcripts (up to 150-fold more than in WT plants) and it restored *SAG12*, *SAG29,* and *SEN4* gene expression to the WT level, but still had somewhat higher transcript levels of *WRKY53* and *WRKY33*, which were approximately 2.9-fold and 2.4-fold higher than that in WT, respectively (Figure 2). 

Thus, ectopic overexpression of the full-length WHY1 and the nuclear isoform nWHY1 had distinguishable senescence-related phenotypes from that of the plastid isoform pWHY1. Yet, at the molecular level, senescence-related gene expression was not always correlated to phenotypes in the 35S promoter-driven overexpression lines, in part probably due to the abnormally higher levels of transgenic expression, which might result in temporary perturbation on gene regulatory networks. Nevertheless, the forced plastid accumulation of WHY1 presumably meant the loss of its nuclear function as a transcriptional regulator.

### 3.2. Allocation of WHY1 into Plastid-Enhanced Production of Reactive Oxygen Species (ROS) 

It was previously reported that the plastid isoform of WHY1 interacted with the light-harvesting protein LHCA1, and had a positive effect on heat dissipation from singlet excited chlorophylls under high light conditions [37], a protective mechanism coupled with the generation of reactive oxygen species [48]. The impressed leaf phenotype of the *pWHY1* overexpression line prompted us to consider a possible link to ROS perturbation. Under normal growth conditions in WT plants, the expression of the senescence-related gene *WRKY53* reached its maximum at 37 days post-germination (Supplementary Figure S2a), while in the early senescence mutant *why1*, its transcripts peaked earlier by approximately one to two weeks (unpublished observation). Peak expression of *WRKY53* was considered as a sign of the initiation stage of leaf senescence [23,43]. In our experiment, the *why1* plants at day 37 also showed a peak in leaf H_2_O_2_ content (Supplementary Figure S2b). Therefore, by using the timing of senescence in WT plants as a reference, we selected this time point to further examine the effects of overexpressed *WHY1* isoforms on ROS status.

**Figure 3:**
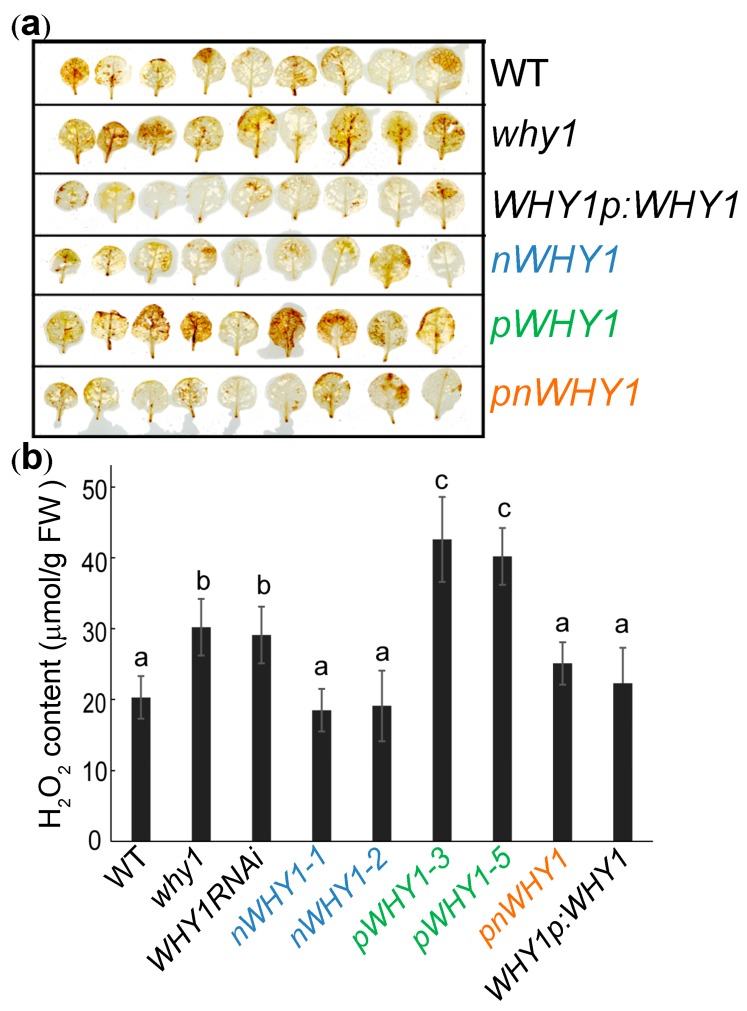
H_2_O_2_ content in leaves of WT, *why1*, and transgenic plants at day 37 post-germination. (**a**) Representative DAB staining of rosette leaves for visualization of H_2_O_2_ accumulation; (**b**) H_2_O_2_ content in rosettes of the plants. Values are shown as mean ± SD (*n* = 3). Bars labeled with the same letter were not significantly different based on one-way ANOVA using the Prism software, *p* < 0.05.

Both DAB staining and quantitative assay confirmed that the *pWHY1* transgenic plants contained the highest H_2_O_2_ in the rosettes compared with the other plants, followed by the loss-of-function mutants, *why1* and *WHY1RNAi* (Figure 3). Overexpression of nuclear isoform nWHY1 in the *why1* background, as well as the full-length WHY1, either driven by the constitute promoter 35S or the native promoter, restored the high H_2_O_2_ accumulation of *why1* plants to a level comparable to that of the WT plants (Figure 3). A similar result was obtained using whole plant staining with DAB and NBT, although the signal difference was not so strong due to sensitivity of the method (Supplementary Figure S3).

### 3.3. PRX33 and PRX39 were Downstream of WHY1 but with no Obvious Involvement in WHY1-Mediated ROS Pathway

**Figure 4:**
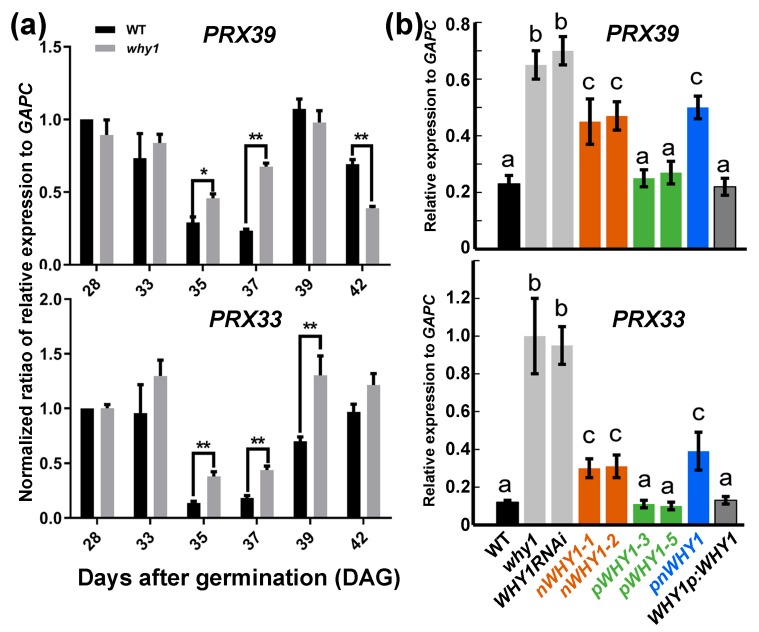
Quantitative RT-PCR analysis of *PRX39* and *PRX33* expression. (**a**) Gene expression during development in WT and *why1* plants. Relative expression was normalized to that of WT at 28 days after germination; significant difference between WT and *why1* plants was determined by pairwise t-test. * *p* < 0.05, ** *p* < 0.01; (**b**) Gene expression in different lines with ectopic overexpression of WHY1 at 37 days after germination. Error bars represent SD for three biological replicates. Bars with the same letter were not significant in the one-way ANOVA test (*p* < 0.05).

Next, to determine whether the expression of genes responsible for the endogenous generation and scavenging of ROS were affected by the overexpression of the WHY1 isoforms, we checked the expression of ten related genes in WT and loss-of-function WHY1 mutant plants during development, from day 28 through day 42 post-germination, when senescence was set. These included genes encoding superoxide dismutase 1 (*SOD1*, cytosolic) and 2 (*SOD2*, chloroplastic), two chloroplastic ascorbate peroxidases (stromal *sAPX* and thylakoid *tAPX*), one transmembrane cytochrome b561/ferric reductase, and peroxidase 33 (*PRX33*) and 39 (*PRX39*), among others. Unexpectedly, only the peroxidase genes *PRX33* (*at3g49110*) and *PRX39* (*at4g11290)* were upregulated in loss-of-function WHY1 plants at days 35 and 37, whereas the expression of the other plants remained unchanged (Figure 4a, Supplementary Figure S4a). Both *PRX33* and *PRX39* belong to class III plant-specific peroxidases that are responsible for apoplastic ROS burst and implicated in cellular growth and in stress signaling in response to numerous biotic or abiotic stimuli [49]. We further compared their expression levels in the WHY1 overexpression lines at the 37th day post-germination.

The transcript levels of *SOD1* and *SOD2* did not differ significantly among all genetic backgrounds (Supplementary Figure S4b). Both *PRX33* and *PRX39* showed similar expression patterns depending on the *WHY1* genetic background (Figure 4b). The loss-of-function WHY1 plants of both the knockout and knockdown lines showed enhanced expression of *PRX33* and *PRX39*, while complementation with full-length WHY1 under its native promoter restored a comparable mRNA level to the WT plants. The 35S promoter-driven full-length WHY1 (*pnWHY1* plants) only partially reduced the transcript levels of both *PRX33* and *PRX39*, which were still higher than in the WT plants. Despite the similar phenotypes and the low H_2_O_2_ content (relative to the WT) in both *WHY1p:WHY1* and *pnWHY1* plants (Figure 3 and Figure 4), the *pnWHY1* plants expressed an unusually higher level of WHY1 transcripts than the WT plants by ~150-fold (Figure 2). This abnormality might account for the subtle difference in the expression of peroxidase genes between both lines, even though no obvious phenotype difference was observed. In the transgenic line of nWHY1, both *PRX33* and *PRX39* gene expression levels were much reduced from that of the *why1* mutant, but comparable to that in the *pnWHY1* plants, and were still higher than that in the WT plants (Figure 4b). Thus, the *PRX33/39* gene expression was affected similarly in *nWHY1* and *pnWHY1* plants. 

Unexpectedly, the high H_2_O_2_-containing *pWHY1* plants showed reduced transcript levels of both *PRX33* and *PRX39* and the levels were closer to that observed in WT (Figure 4b). In this case, PRX33/39 may not contribute to H_2_O_2_ generation, or the high H_2_O_2_ content in the transgenic plants had a feedback effect inhibiting the expression of both genes. 

These observations indicated that the elevated expression of *PRX33* and *PRX39* in loss-of-function WHY1 lines could be further reduced by ectopic expression of WHY1, though only the full-length WHY1 under a native promoter control could restore the expression to the WT level (Figure 4b), suggesting that WHY1 might work as their transcriptional upstream regulator and that a proper amount of the nuclear isoform nWHY1 might be necessary for repression.

**Figure 5:**
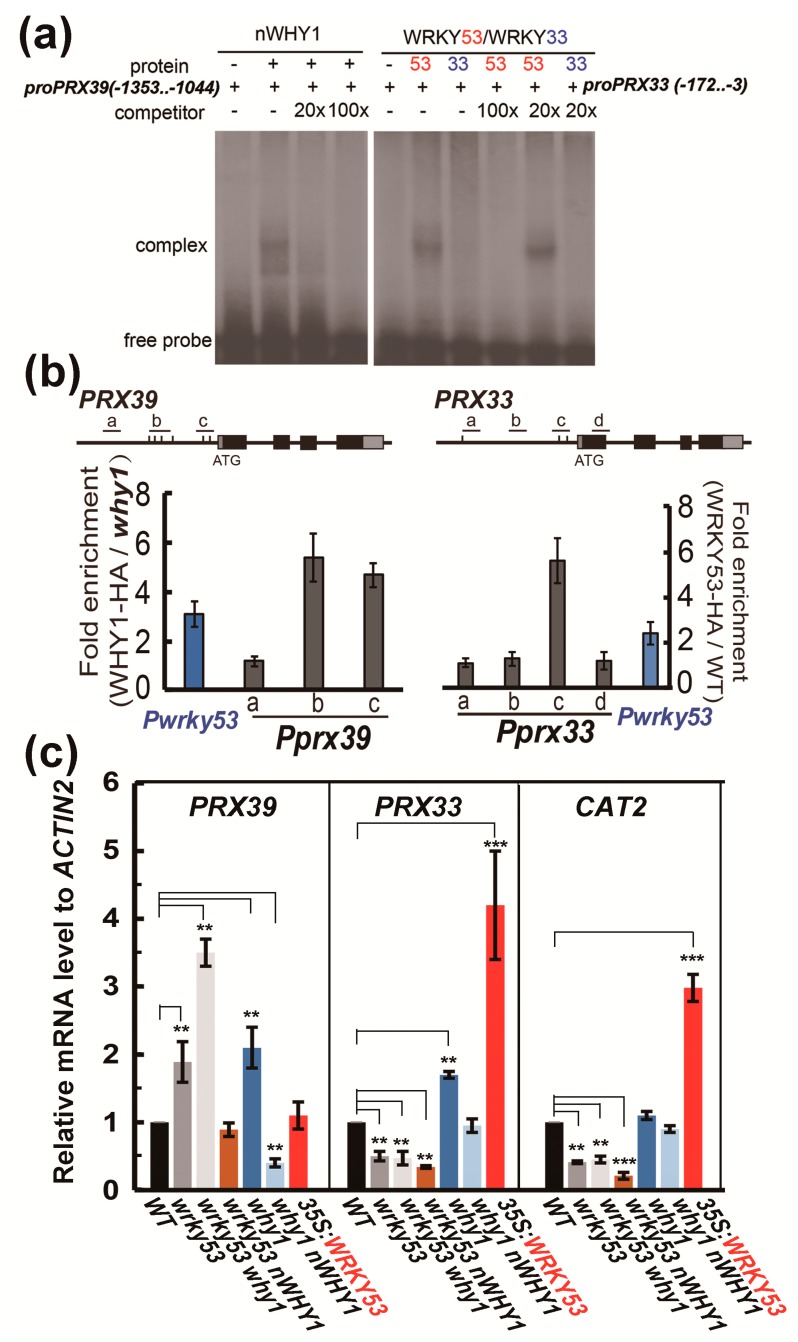
Transcriptional regulation of *PRX39* and *PRX33* by *WHY1* and *WRKY53*. (**a**) Electrophoretic mobility shift assay demonstrating the binding of WRKY53 to 5′-UTR and promoter fragment of *PRX33* and the binding of WHY1 to a region of *PRX39* promoter (+1 for start codon, see Materials and Methods for details); (**b**) Chromatin immunoprecipitation quantitative PCR (ChIP-qPCR) determination of the enrichment of *PRX39* or *PRX33* promoter sequences associated with WHY1-HA (left panel) or WRKY53-HA (right panel). Anti-HA antibody was used to precipitate the cross-linked genomic DNA fragments in which the number of designated regions was compared to that in the non-precipitated input DNAs to determine the enrichment factors. Fold enrichment was calculated by comparing the enrichment in overexpression line to that in the background line. The fold enrichment of *WRKY53* promoter fragments was shown in both cases for an experimental control, since *WRKY53* was a known transcriptional target of WHY1 and itself. Error bars represent standard errors for three biological replicates. (**c**) Both *PRX33* and *PRX39*, as well as the senescence-related catalase gene *CAT2*, were expressed differently in several mutant and transgenic plants for *WRKY53* and *nWHY1*. Values were shown as the means of three biological replicates. Asterisks indicate significant differences from the WT according to two-tail Student’s t-test (* *p* < 0.05, ** *p* < 0.01, and *** *p* < 0.001).

A further analysis of the promoter sequence by using the PlantCARE Program (http://bioinformatics.psb.ugent.be/webtools/plantcare/html/) [50] predicted the existence of two W-box elements in the promoter of *PRX33* and several WHY1-binding elements (including GTNNNAAATT) in the promoter of *PRX39*, suggesting that *PRX33* and *PRX39* might be a target downstream of WRKY53 and WHY1, respectively. To this end, we performed electrophoretic mobility shift assays (EMSAs), and confirmed that the purified recombinant WRKY53 physically interacted with a 169 bp, two W-box-containing fragment in the *PRX33* promoter region (-3 upstream from the star codon), whereas the negative control protein WRKY33 did not have the ability to interact with the DNA fragments (Figure 5a, right panel). Similarly, the recombinant WHY1 protein could interact with a 309 bp fragment from the *PRX39* promoter region containing two consensus GTNNNAAATT elements in the EMSA assay (Figure 5a, left panel). The binding of WHY1 on *PRX33* promoter DNA and the binding of WRKY53 on *PRX39* promoter DNA were further confirmed by in planta chromatin immunoprecipitation quantitative PCR (ChIP-qPCR) experiments, in which transgenic lines expressing the HA-tagged WHY1 and WRKY53 were used as plant materials. In the WHY1-HA-expressing plants, the two GTNNNAAATT fragments within the promoter of *PRX39* were significantly enriched by immunoprecipitation with an antibody against the HA tag, by approximately 4.8-fold and 5.5-fold compared to that in the *why1* plants (Figure 5b, left panel). As a comparison, the promoter GTNNNAAATT fragments of *WRKY53* were enriched by a factor of 3.5 over the mock. In the WRKY53-HA-expressing plants, the WRKY53-binding W-box region in *PRX33* promoter was enriched by about 5.4-fold compared to that in the WT plants (without a tagged WRKY53), while the promoter W-box region in *WRKY53,* known to interact with its own protein, was also enriched by a factor of 2.4 over that in the mock plants (Figure 5b, right panel).

These experiments established that WHY1 worked as a transcriptional repressor of *PRX39*, and indirectly affected *PRX33* expression via inhibiting its upstream gene *WRKY53* [23].

Next, we further checked their expression levels in a number of mutant lines with *wrky53* or *why1* background (Figure 5c). The *PRX39* transcript levels were raised by a loss of function of both *WRKY53* and *WHY1*, and even more so in the double mutant (Figure 5c). The overexpression of *WRKY53* in a WT background or the overexpression of the nuclear isoform of *WHY1* in a *wrky53* background conferred a similar transcript level of *PRX39* as observed in the WT plants. In the *why1* plants overexpressing the nuclear isoform of WHY1, *PRX39* expression was significantly reduced to more than half of that detected in the WT plants (Figure 5c). Therefore, *PRX39* was negatively regulated by both WHY1 and WRKY53 at the transcriptional level under the tested conditions. Although the mechanism of how WRKY53 repressed *PRX39* was not known for the moment, nevertheless, it seemed that the loss of WHY1 might have two opposite effects in regulating *PRX39* gene expression: (i) de-repression on *PRX39* that was negative and (ii) de-repression on WRKY53 that was positive.

The transcript level of *PRX33* was oppositely influenced by loss-of-function mutants of *WRKY53* and *WHY1*, its upregulation could result from an overexpression of *WRKY53* or a loss of *WHY1* (Figure 5c). Since WHY1 was a repressor of *WRKY53*, loss of WHY1 might actually lead to accumulated WRKY53 proteins that in turn activate *PRX33*. In consistence with this, plants of *wrky53*, *wrky53 why1*, and *wrky53 nWHY1* mutant lines showed a similar *PRX33* mRNA level (Figure 5c). Furthermore, overexpression of *nWHY1* in the *why1* background reduced the elevated transcript level of *PRX33* caused by loss of *WHY1* (Figure 5c). Thus, *PRX33* was an indirect downstream target of WHY1, likely in part via inhibition of its activator gene *WRKY53*. A similar situation was found in the senescence-related catalase gene *CAT2* (Figure 5c), which was a known target of *WRKY53* [51].

Although WHY1 was able to repress *PRX39* and *PRX33* via direct or indirect transcriptional regulation, involvement of both peroxidase genes in WHY1-mediated ROS/H_2_O_2_ pathways could not be revealed by the present experiment. Furthermore, their loss-of-function mutations displayed a moderate phenotype of early senescence as compared to the WT plants (Supplementary Figure S3), indicating that they might have positive functions against senescence. The homozygous T-DNA insertion line of *prx39* had an early senescence phenotype similar to plants of *pWHY1* overexpression, whereas the homozygous *prx33* T-DNA mutant showed a moderate senescence phenotype in the *nWHY1* overexpression lines and the *pWHY1* plants (Supplementary Figure S3, upper panel). Accumulations of ROS and H_2_O_2_, observed by NBT and DAB staining, respectively, were stronger in the *prx39* plants, but weaker in the *prx33* plants (Supplementary Figure S3, lower panel). These observations suggested that *PRX39* and *PRX33* might be required for proper maintenance of ROS/H_2_O_2_ homeostasis during senescence. Nevertheless, WHY1 repression of senescence might necessitate the ROS/H_2_O_2_ pathway in cells, which involved other unknown factors not limited to *PRX33* and *PRX39*.

### 3.4. H_2_O_2_ Treatments Altered WHY1 Protein Distribution Between Plastids and the Nucleus but Not Its mRNA Levels 

**Figure 6:**
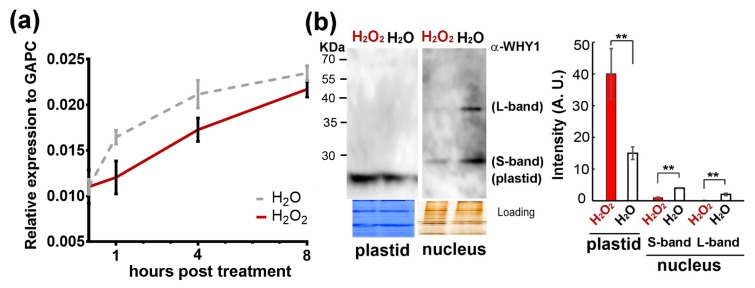
Effects of exogenous H_2_O_2_ treatments on transcript levels and protein subcellular distributions of *WHY1* in 5-week-old WT plants. (**a**) Relative gene expression levels in plants sprayed with 0.3% H_2_O_2._ Error bars represent the SD of three biological replicates. No significant differences were detected using a multiple t-test by the two-stage linear step-up procedure of Benjamini et al. in GraphPad Prism (version 7.1); (**b**) Organelle isoforms of WHY1 proteins in WT plants receiving H_2_O_2_ or H_2_O treatment. Left, a representative protein immunodetection of purified plastid and nuclear fractions at 4 h post-treatment. The purity of nuclear and plastid proteins without cross-contamination was monitored using antibodies against histone 3 or photosystem II (PSII) protein (Supplementary Figure S5). Coomassie and silver staining were used as loading controls, and the western blot was detected using an anti-WHY1 antibody. Right, quantitative measurement of protein band intensity using Image J software (see Materials and Methods for details). Values were means of three independent experiments. Statistical significance was analyzed using a paired Student’s t-test. **p < 0.01.

We further determined how *WHY1* gene expression responded to H_2_O_2_ treatment, since an elevation in endogenous ROS was known as a cell death signal and might lead to plant senescence [52,53]. Upon H_2_O_2_ treatment, *WHY1* transcripts were slightly lower than in the water control, with a similar pattern of expression during the 8-hour period post-treatment (Figure 6a). These small differences were not significant. We then reasoned that the treatment might instead affect the WHY1 protein distribution between plastids and the nucleus. Using isolated nucleus and plastid fractions from 5-week-old WT rosettes, we conducted western blotting with a specific antibody against WHY1 (Supplementary Figure S5). Two bands in the purified nucleus fraction, corresponding to the large (~37 kDa) and small (~29 kDa) nuclear WHY1 isoforms, and one band in the plastid fraction (~24 kDa) were detected; these WHY1 isoforms were denoted as ‘L-band’, ‘S-band’, and ‘plastid’ in Figure 6b, respectively. The full-length AtWHY1 protein had a putative molecular weight of ~29 kDa, the N-terminal chroloplast transit peptide (CTP) (~47aa) may be removed once imported into the plastids. This truncated form had a predictive molecular weight of ~24 kDa, consistent with the plastid band in Figure 6b. The S-band was close to the predicted full-length (~29 kDa), while the L-band (~ 37 kDa) seemed to be a modified form or bound by other unknown small peptides. 

Four hours after H_2_O_2_ treatment, both L- and S-bands of the nuclear isoforms were greatly reduced in quantity, while the plastid isoform accumulated significantly. Particularly, the L-band, which represented a putatively modified isoform, almost disappeared (Figure 6b). Thus, external application of H_2_O_2_ had a major effect on WHY1 protein isoforms shifting to the plastids, without a significant change in its gene expression.

### 3.5. H_2_O_2_ Induces the Enrichment of H3K9ac and RNAP II at WRKY53 Promoter Region

**Figure 7:**
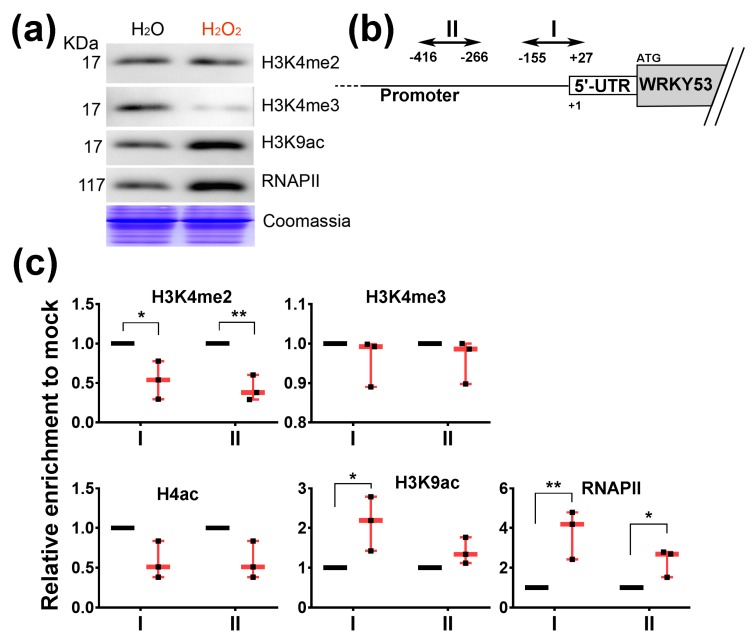
Detection of global and *WRKY53*-locus-specific H3K4 methylations, H3K9 acetylation, and RNAP II recruitment in 28-day-old plants at 4 h post-treatment with H_2_O or H_2_O_2_. (**a**) Global immunodetection on isolated total proteins using indicated specific antibodies; (**b**) Schematic dispatch of the promoter and 5′-UTR region in *WRKY53* locus, showing the location of the two detection PCR amplicons (I and II); (**c**) ChIP-PCR determination of relative enrichment of DNA at I and II of the *WRKY53* locus, shown as normalized ratios of ChIP/input to that of the H_2_O treatments (mock, in three biological replicates). Individual data for three biological replicates normalized to their respective mock treatments were plotted, with minimum to maximum bars and the medium points in red. Asterisks indicate significant differences from mock using a paired Student’s t-test. * *p* < 0.05; ** *p* < 0.01.

Alterations in H3K4me3 and H3K9ac globally [54] and specifically at the *WRKY53* locus [23,27,55] had been observed during plant senescence. The loss of nuclear isoforms of WHY1 upon exogenous H_2_O_2_ treatment prompted us to check whether specific or global histone modification occurred under this condition. Western blots revealed that the bulk of H3K4me2 markers were not changed, but global H3K4me3 was blocked and total H3K9ac and RNA polymerase II (RNAP II) increased greatly after H_2_O_2_ treatment (Figure 7a). At the *WRKY53* locus, enrichment of H3K4me2 at two regions in the 5′-UTR and promoter was significantly reduced in H_2_O_2_-treated plants, by more than half of that in the mock-treated plants, although such effect was not found for H3K4me3 (Figure 7b,c). These two regions had a high recruitment ratio for RNAP II upon H_2_O_2_ treatment, by 4-fold and 3-fold over the mock (Figure 7c). Furthermore, enrichment of histone 4 acetylation at the regions was reduced insignificantly, but H3K9ac increased by more than 2-fold after H_2_O_2_ treatment (Figure 7c). Taken together, these results indicated that a decrease of WHY1 nuclear isoform caused by exogenous H_2_O_2_ treatment was coincident with a global reduction in H3K4me3 markers, and it also induced the expression of H3K9ac markers and the recruitment of RNAP II globally and specifically at the *WRKY53* promoter. 

## 4. Discussion

The idea that WHIRLY proteins might be associated with retrograde signaling had been suggested for many years; however, strong evidence was still missing. In a previous study, we reported that the WHY1 protein was phosphorylated by a CIPK14 kinase, shifting to its nuclear localization and altering its cellular functionalities between plastids and the nucleus [20]. Here, we further revealed that the dual-location of WHY1 protein was linked to a perturbation in H_2_O_2_ homeostasis and thus might intervene as parts of a retrograde connection between plastids and the nucleus. The loss of *WHY1* enhanced H_2_O_2_ accumulation at 37 days post-germination and was associated with an early senescence phenotype. In line with that, complementary expression of *WHY1* driven by the native promoter restored the mutant’s H_2_O_2_ level comparable to that of the WT. The rising production of H_2_O_2_ during senescence could be suppressed by specific ectopic expression of the nuclear isoforms of WHY1, but enhanced by the plastid isoforms (Figure 3). The underlying mechanism, however, was still elusive from the current studies. Although we showed that WHY1 was able to bind to the promoter of peroxidase gene *PRX39* and repress its expression, and inhibited another peroxidase gene (*PRX33*) via repressing its activator gene *WRKY53* (Figure 5), whether both peroxidases were involved in WHY1-mediated process was yet questionable. Given the fact that the intracellular levels of H_2_O_2_ were tightly controlled by a comprehensive inventory of both H_2_O_2_-generating systems and antioxidant proteins [56], it was reasonable that *PRX33* and *PRX39* were not the only players for cellular H_2_O_2_ homeostasis [49,57]. In addition, other pathways might also contribute to the regulatory network during senescence, either acting independently or jointly with the WHY1 axis. Examples of these include the salicylic acid and abscisic acid (ABA) pathways [53,58,59,60]. Interestingly, PRX33 protein was found among the H_2_O_2_-detoxifying proteins that had their expression levels altered, in a light-responding way or an age-dependent manner, by the so-called plastid transcription active chromosome (TAC) proteins. The plastid isoform of WHY1 was a component of the plastid TAC [28], which contained a total of 35 nuclear- or plastid-encoded proteins. Furthermore, PRX33 protein was reported by proteome analysis to be enriched in the *why1why3* double mutants [61]. The homologous WHY3 shared 78% and 82% amino acid identity and similarity with WHY1, respectively, and was putatively dual-localized [17]. Although the real function of WHY3 was not yet clear, it was believed that WHY3 worked synergistically as a cofactor of WHY1, and was involved in stabilizing photosystem I and balancing ROS homeostasis [33,37,62]. In our experiments, the abnormal transcript levels by ectopically expressed WHY1 isoforms (Figure 2) might affect the interactions between *WHY1* and *WHY3*, possibly at the mRNA level or the protein level. Of course, that would definitely require further evaluation.

It is also worth to note that in our construct for pWHY1 overexpression, a nucleus exist sequence was included at the C-terminal of WHY1, which might possibly lead to cytosol accumulation of WHY1 proteins and contributed in some ways to the observed phenotype.

On the other hand, an elevation of H_2_O_2_ level enhanced plastid accumulation of WHY1 and decreased the nuclear isoform (Figure 6), leading to H3K9ac enrichment and RNAP II recruitment at the *WRKY53* promoter (Figure 7). This observation confirmed earlier results that pre-toxic H_2_O_2_ was an important signal molecule during senescence [23,37] or in response to biotic and abiotic cues [36,63]. Thus, a shift of WHY1 proteins from the nucleus to plastids was a likely reflection of the cellular stresses signaled by H_2_O_2_. Since the distribution of WHY1 to the nucleus depended on its phosphorylation by CIPK14 kinase [20], it would be interesting to see how H_2_O_2_ interplays with the kinase network. Furthermore, our immunodetection revealed that the purified nuclear isoform of WHY1 proteins appeared as two bands on the western blot (~37 kDa and ~29 kDa, Figure 6b). This was similarly observed in barley (unpublished, see also [18]), in which the large band was proposed as a modified form under some unverified conditions. The ~29 kDa band met well with the predicted MW of the full-length protein and was slightly larger than a plastid form with a truncated CTP signal sequence (~24 kDa), therefore, it did not seem to be translocated from plastid to the nucleus, as proposed previously [19]. Thus, it was more likely that the WHY1 protein had two separated isoforms for the plastid and the nucleus, and both were subjected to different modifications in their compartments under stress conditions. 

**Figure 8:**
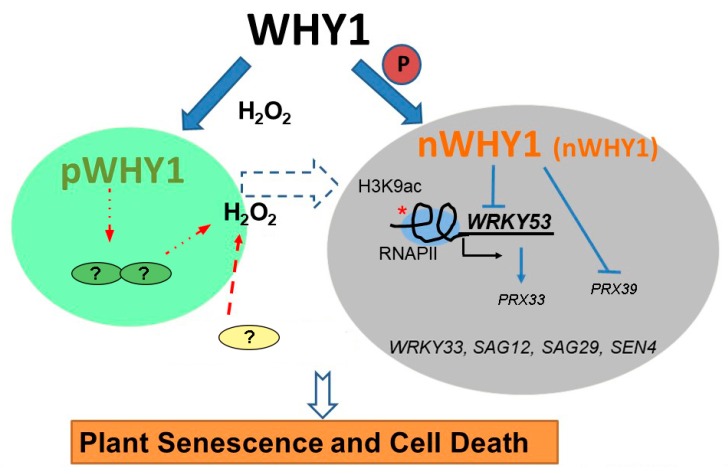
A working schema of the senescence pathway performed by the dually located WHY1 in combination with H_2_O_2_. The nuclear isoform of WHY1 is represented as both a large molecular mass protein (L-band, bigger letters) and a small molecular mass protein (S-band, smaller letters). The WHY1 has dual functions in plastids and the nucleus. Loss of WHY1 or shifting the proteins to plastids increases H_2_O_2_ accumulation through an unknown pathway, resulting in a senescence phenotype. Elevated H_2_O_2_ represses nuclear WHY1 accumulation, promoting H3K9ac enrichment and RNAP II recruitment globally and specifically at the *WRKY53* locus, and stimulating early senescence. Thus, distribution of WHY1 organelle isoforms and the putative feedback of H_2_O_2_ form a circularly integrated regulatory network during plant senescence in *Arabidopsis*. Plastid is shown as a green ovary, nucleus as a grey ovary, lines for regulation, fat arrows for transfer or translocation, and broken lines for uncertainty.

In conclusion, by using a series of mutants and transgenic plants with targeted expression of organelle isoforms of WHY1, we have demonstrated that the allocation of this dually located protein between plastid and nucleus has disparate effects on plant senescence. The nuclear isoform of WHY1 is the authentic repressor of several senescence-associated genes, including *WRKY53*, *WRKY33*, *SAG12*, *SAG29,* and *SEN4*, as well as two ROS-related peroxidase genes *PRX39* and *PRX33*, the involvement of which are not clear yet. A shift from nucleus to plastid isoform promotes H_2_O_2_ accumulation and accelerates plant senescence. This shift may occur during natural aging or may be caused by elevated H_2_O_2_, generated from diverse organelles during cellular metabolism or produced under abiotic/biotic stresses. Thus, H_2_O_2_ serves as a potential feedback signal by altering the subcellular distribution of WHY1 to enforce plant senescence and cell death (Figure 8).

## Supplementary Materials

The following are available online at https://www.mdpi.com/2073-4409/8/12/1585/s1. Supplementary Figure S1. Verification of *WHY1* transgenic plants in transcript and protein level by semi-RT-PCR and western blot; Supplementary Figure S2. Time course expression of senescence marker genes and H_2_O_2_ content in WT plants and *why1* plants during plant aging; Supplementary Figure S3. Representative images showing senescence-related phenotypes of *prx33* and *prx39* mutant plants in comparison with *why1* and their transgenic plants; Supplementary Figure S4. Gene expression during six weeks post-germination in WT and *why1* plants; Supplementary Figure S5. Western blot detection of specificity of an antibody against WHY1 peptide (A-B) and purity of nuclear protein, plastid protein (C); Supplementary Table S1. List of primer sequences for PCR.
