# Supplementary material for: H2O2 as a Feedback Signal on Dual-Located WHIRLY1 Associates with Leaf Senescence in Arabidopsis
Source: Cells. 2019 Dec 6;8(12):1585. doi: 10.3390/cells8121585 (PMC6952816; doi:10.3390/cells8121585)
Supplement: Supplementary file 1 [file cells-08-01585-s001.zip › Supplementary table S1 The list of primer sequences for PCR.pdf]

Supplementary Table S1. The list of primer sequences for PCR

Gene loci	Gene name	Primer sequences
**For qRT-PCR**		
at1g13440	*AtGAPC2*	FP: 5'-ACCACTGTCCACTCTATCACTGA-3'<br>RP:5'-TGAGGGATGGCAACACTTTCCC-3'
at5g45890	*SAG12*	FP: 5'-ACAAAGGCGAAGACGCTACTTG-3'<br>RP:5'-ACCGGGACATCCTCATAACCTG-3'
at5g13170	*SAG29*	FP: 5'-GCCACCAGGGAGAAAAGG-3'<br>RP:5'-CCACGAAATGTGTTACCATTAGAA-3'
at4g30270	*SEN4*	FP: 5'-AAGGTGACAAAGAGCAACAATTC-3'<br>RP: 5'-CTCTCTAATGGGTGTGTCATCG-3'
at2g38470	*WRKY33*	FP: 5'-CTTCCACTTGTTTCAGTCCCTCTC-3'<br>RP:5'-CTGTGGTTGGAGAAGCTAGAACG-3'
at4g23810	*WRKY53*	FP: 5'-GACGGCTGTTGCTGAGACTA-3'<br>RP: 5'-ATTTGCCACCGGGACTACAG-3'
at3g49110	*PRX33*	FP: 5'-ACAATCTGTCACTTTGGCAGGAG-3'<br>RP: 5'-AGAATGGAGCTGGAAGATTTGCG-3'
at4g11290	*PRX39*	FP: 5'-TTATCCGGTGCCCACACGATTG-3'<br>RP: 5'-AACGCCGGTGAAATTGAAGAGAC-3'
at1g08830	*SOD1*	FP: 5'-AACGGTTGCATGTCTACTGGTC-3'<br>RP: 5'-GTGATTGTGAAGGTGGCAGTTCC-3'
at2g28190	*SOD2*	FP: 5'-CAGGGCCTCATGGATTTCATCTCC-3'<br>RP: 5'-TGGAGCTCCGTGTGTCATGTTG-3'
at1g14410	*WHIRLY1*	FP: 5’-TTTTACGTGGGTCATTCGAT-3’<br>RP: 5’-GTCCACTGTTAACGCAGCTT-3
at4g35090	*CAT2*	FP: 5’-TTTGCAGAGAATGAGCAACTTG-3’<br>RP:5’-TATCTCAACTTTCATCTCTTCCC-3’
**For EMSA DNA fragments of promoter region**		
at3g49110	*PRX33*	FP: 5’- GGTAACCCAAGTATTTTTCA-3’<br>RP: 5’-TCACAAGGACAATGAAAAC-3’
at4g11290	*PRX39*	FP: 5’- GGCAAGTTG GATAAATACA-3’<br>RP: 5’- TCTCAATATTAACTATTAGG-3’
**For ChIP-qPCR**		
at4g23810	*WRKY53 I*	FP: 5’-GATCATATCATTCTTTCAGTCCATC-3’<br>RP: 5’-CCAACTTAACATATCTCTTCCTTCC-3’
at4g23810	*WRKY53 II*	FP: 5’-TCAAACACTGAAAATCCAATGCCT-3’<br>RP: 5’-AATGAGAGTGTTATAGGATTGGTGA-3’
at4g11290	*PRX39a*	FP: 5’-CGTAACTACATTCGTCAAGA-3’<br>RP: 5’-CATGGCATGTGACGTATTTT-3’
	PRX39b	FP: 5’- AGTTCTCGAGAAAATGTAGG-3’<br>RP: 5’-TTAATCATCATCCATTTATC-3’
	PRX39c	FP: 5’- GATAATTGAAATCAAACAGG-3’

		RP: 5’-GGGATTATATATAGTGTTGG-3’
at3g49110	*PRX33a*	FP: 5’- GAACTAATGACGAGAGATCA-3’<br>RP: 5’-AGAAAACTTCTACGACCCTC-3’
	PRX33b	FP: 5’- GATGGCGAAAGCTGGTATTA-3’<br>RP: 5’-GTGAGAGTTATTGTAGGAGAG-3’
	PRX33c	FP: 5’- CATGTACGTAATAATATTCA-3’<br>RP: 5’-AGGCAAAACGGGTCAAACGT-3’
	PRX33d	FP: 5’- ACTTACCCCTACTTTTTACGACACT-3’<br>RP: 5’-GGGTCCGATCTTAGCTCGTTG-3’
**For homozygous screening of T-DNA insertion lines**		
at3g49110,<br>SALK_062314	*prx33*	LP TCAACGGGAGTGTTTTGAATG<br>RP ACGACACTTCATGTCCTACCG
at4g11290,<br>SAIL_757_G03	*prx39*	LP TCAACGATTTTCACAGACGTG<br>RP AAGTGGAGAAGCTTGCTCCTC
