# Supplementary figures and images for: H2O2 as a Feedback Signal on Dual-Located WHIRLY1 Associates with Leaf Senescence in Arabidopsis
Source: Cells. 2019 Dec 6;8(12):1585. doi: 10.3390/cells8121585 (PMC6952816; doi:10.3390/cells8121585)

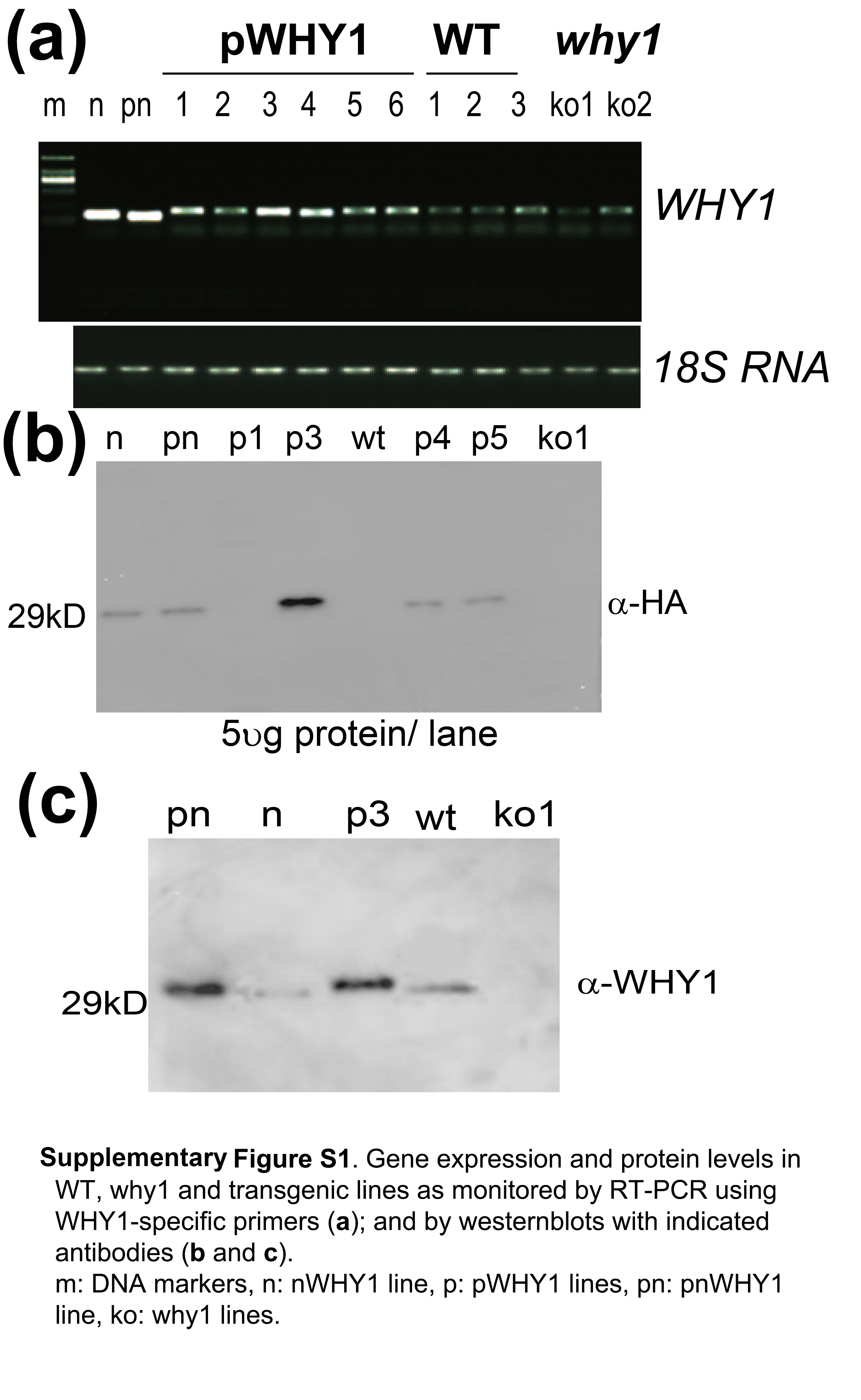

Supplement: Supplementary file 1 [file cells-08-01585-s001.zip › Supplemental Figure S1.tif]

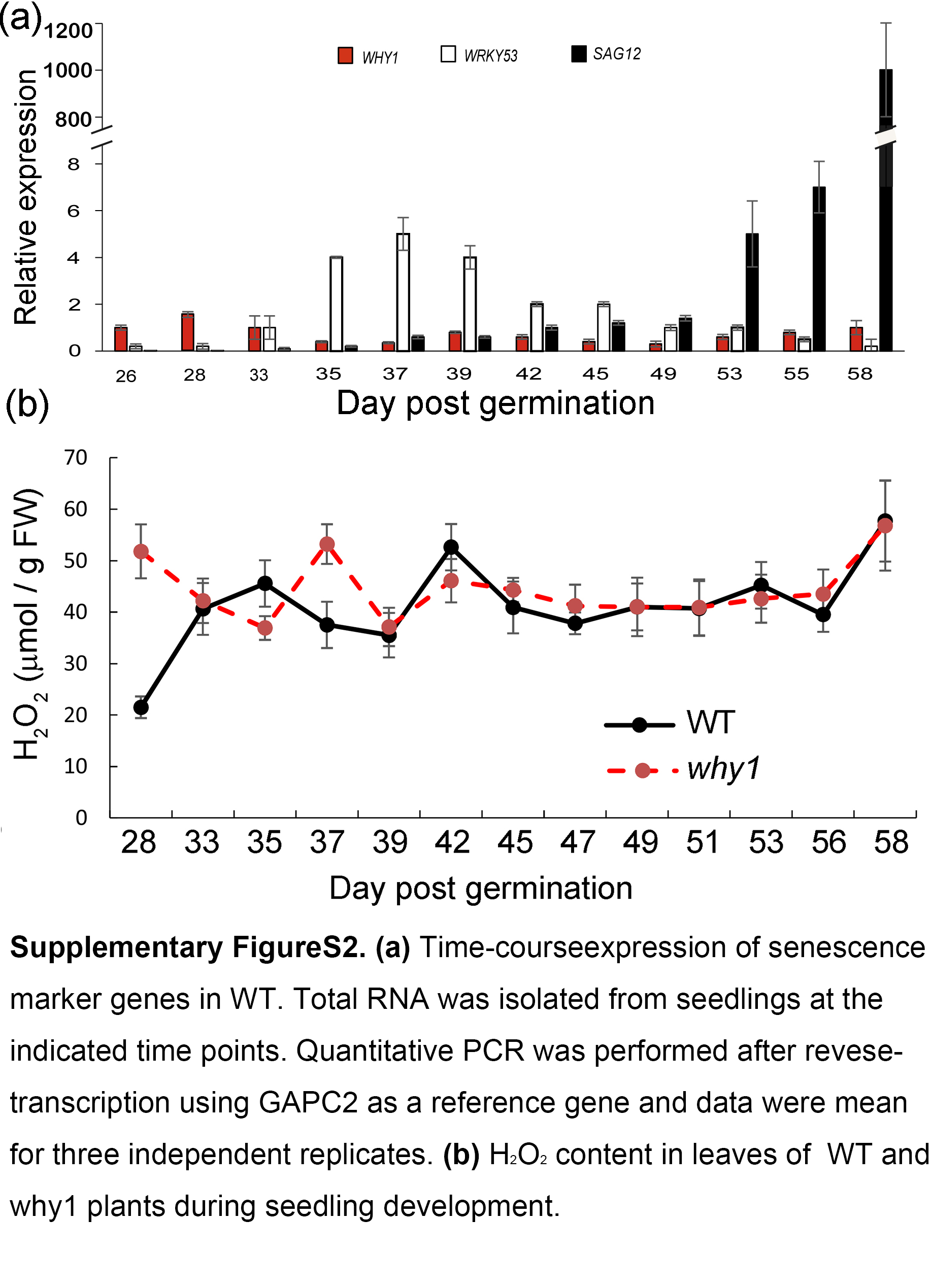

Supplement: Supplementary file 1 [file cells-08-01585-s001.zip › Supplemental Figure S2.tif]

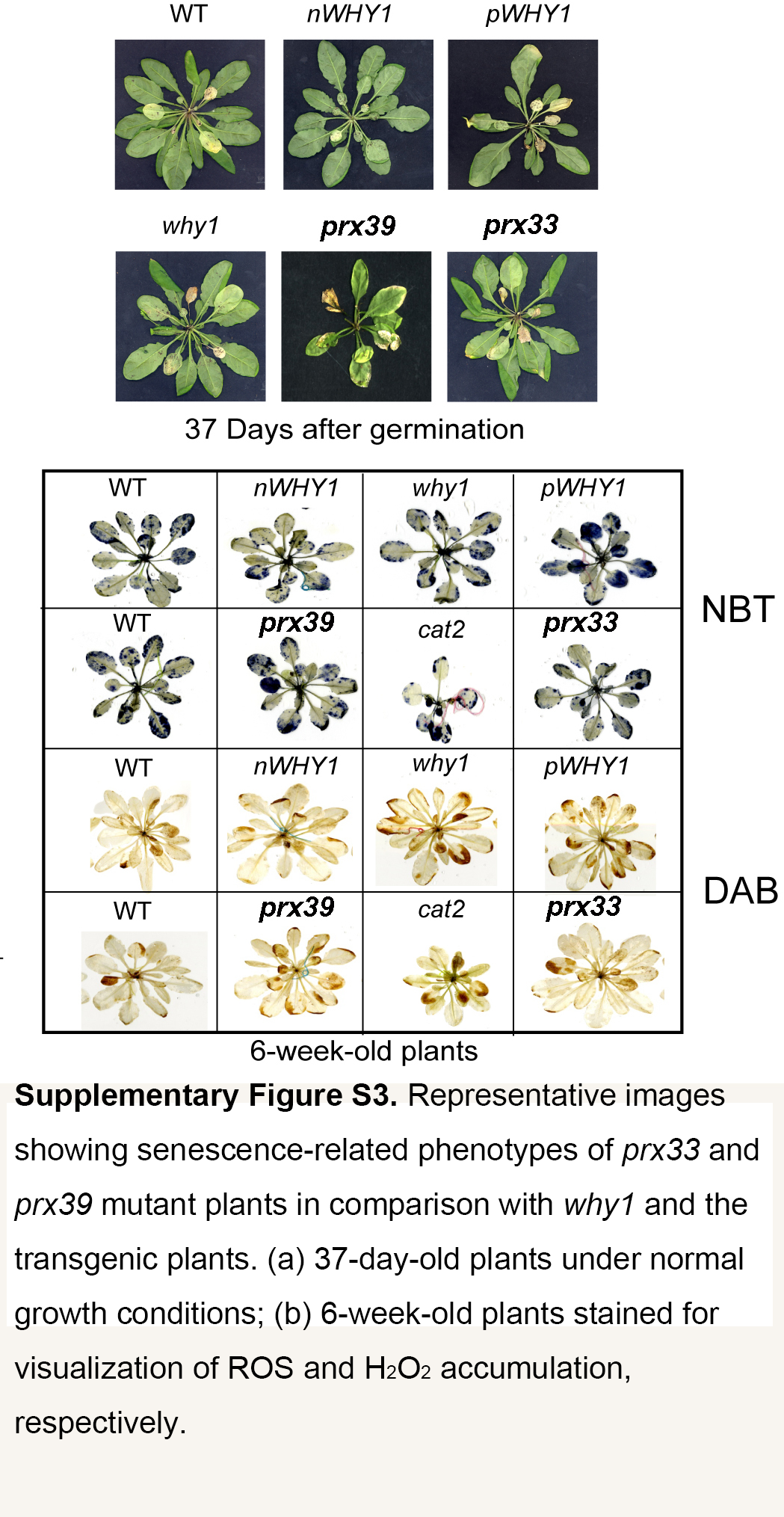

Supplement: Supplementary file 1 [file cells-08-01585-s001.zip › Supplemental Figure S3.tif]

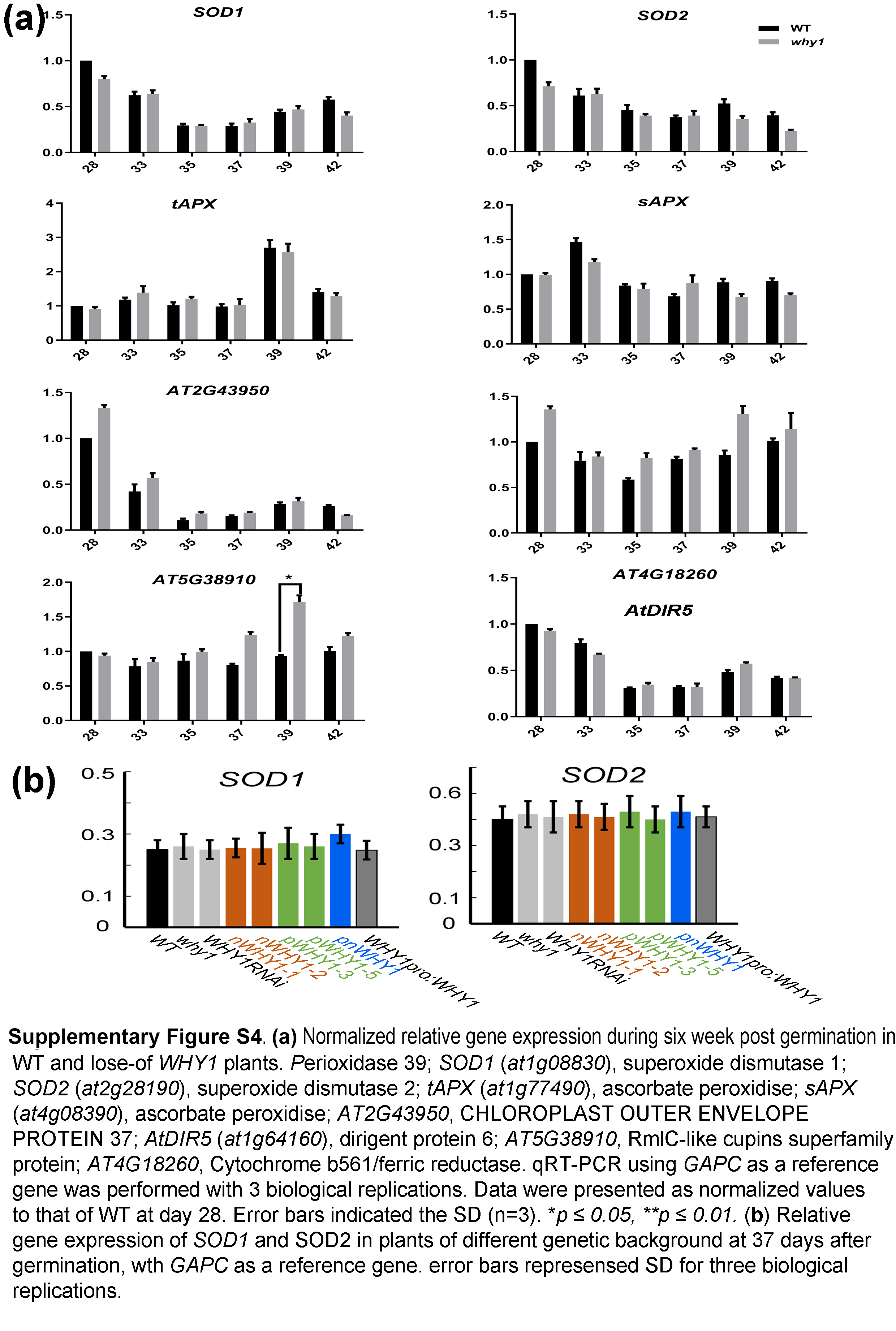

Supplement: Supplementary file 1 [file cells-08-01585-s001.zip › Supplemental Figure S4.tif]

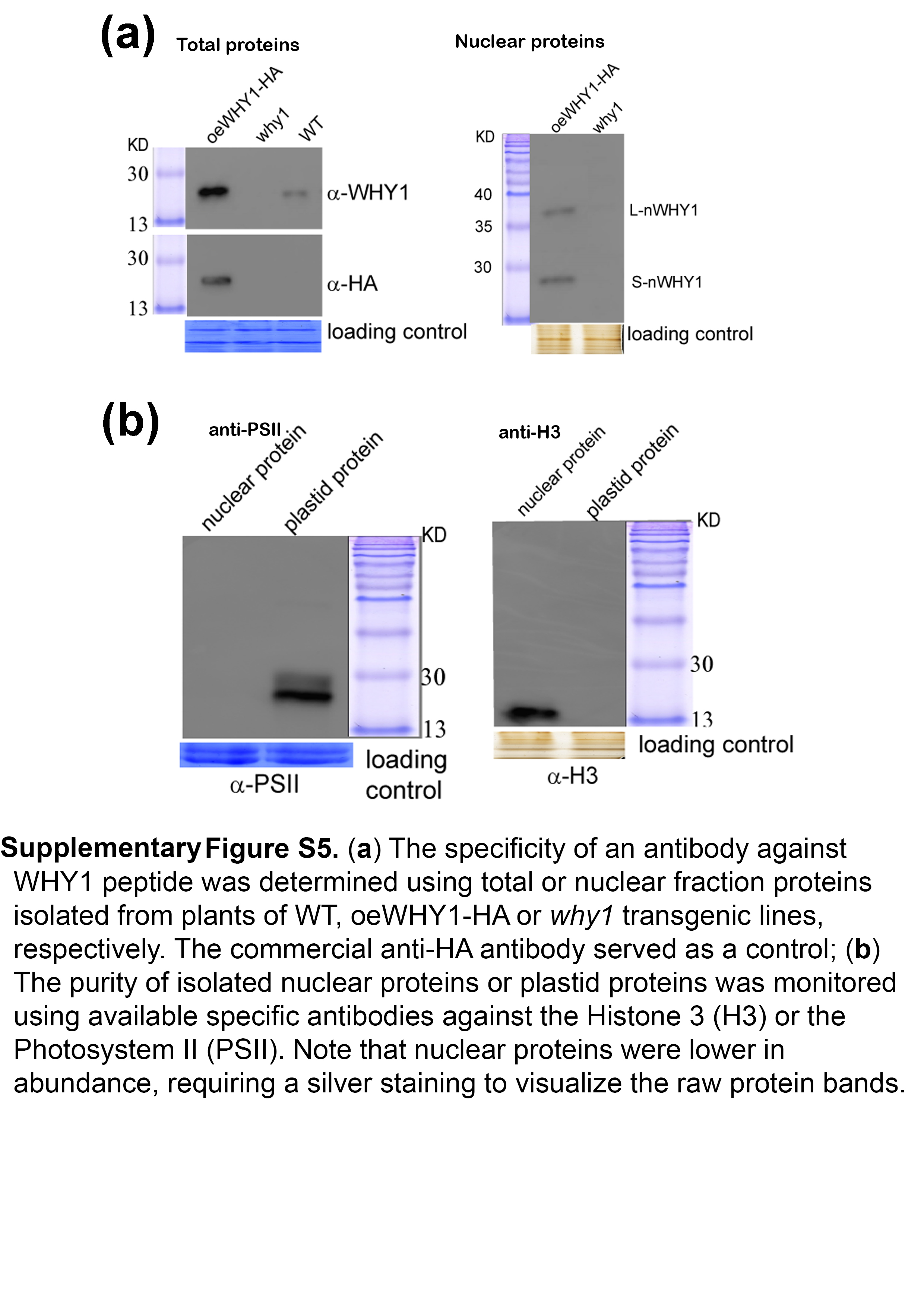

Supplement: Supplementary file 1 [file cells-08-01585-s001.zip › Supplemental Figure S5.tif]
